# Evaluating the Efficacy and Safety of Pluvicto in Chemotherapy-Ineligible Nonagenarians: A Descriptive Case Series

**DOI:** 10.7759/cureus.79119

**Published:** 2025-02-16

**Authors:** Jacob Charron, Kevin Maupin, Chrystal Su, Brandon Mancini, Harshad Kulkarni

**Affiliations:** 1 Theranostics, BAMF Health, Grand Rapids, USA; 2 Radiology, Michigan State University College of Human Medicine, East Lansing, USA; 3 Nuclear Medicine, Keelung Chang Gung Memorial Hospital, Keelung City, TWN

**Keywords:** ineligible for chemotherapy, metastatic castration resistant prostate cancer, nonagenarian population, pluvicto, psma imaging and therapy

## Abstract

Nonagenarians with metastatic castration-resistant prostate cancer (mCRPC) are often underrepresented in prostate cancer research due to their limited numbers among treated populations. For this group, traditional chemotherapy carries significant side effects that can severely impact quality of life. Targeted therapy designed to selectively bind to prostate cancer cells expressing the prostate-specific membrane antigen (PSMA) presents a promising alternative, particularly for those with widespread metastatic disease and contraindications to chemotherapy. This retrospective case series evaluated the response, side effects, and quality of life of three nonagenarian patients with mCRPC who received Pluvicto (177Lu-PSMA-617/177Lu-vipivotide tetraxetan) as standard care at BAMF Health in Grand Rapids, Michigan, USA. Prior to treatment, all patients underwent baseline PSMA PET/CT to confirm PSMA-expressing disease and assess eligibility. They then received standard cycles of 200 mCi Pluvicto every six to eight weeks, followed by a 24-hour single photon emission computed tomography (SPECT)/computed tomography (CT) scan to assess uptake and response. Blood work, including prostate-specific antigen (PSA) levels and a comprehensive review of hematology and chemistry parameters, was conducted before treatment and every two to three weeks. Total-body PSMA-positron emission tomography/CT with Illucix (Ga-68 PSMA-11/Ga-68 gozetotide) was performed for initial assessment and at restaging as needed. All patients showed substantial reductions in tumor burden, as measured by PSA levels and SPECT/CT. Two patients experienced a >70% reduction in PSMA-expressing tumor volume, with PSA nadirs ranging from an 84% to 98% reduction from baseline. Adverse events were mild, including low-grade xerostomia in two patients and a progression from grade 1 to grade 2 anemia in two patients. Only one patient did not complete all six cycles due to disease progression after the fourth cycle. Pluvicto appears to be a promising treatment option for medically fragile nonagenarians with mCRPC. In this case series, the therapy demonstrated strong clinical and radiographic responses, including significant PSA reductions, underscoring its potential efficacy in this population. Its favorable safety profile and minimal toxicities further support its use in these medically vulnerable patients. However, larger studies are needed to enable a more comprehensive analysis and establish the statistical significance of these findings.

## Introduction

Prostate cancer remains the most prevalent malignancy among males, with an estimated 299,010 new cases projected in 2024 and approximately 35,250 associated deaths [1]. Between 2014 and 2019, the incidence of prostate cancer in men aged 75 and older increased by 4.6% annually [2]. Data from the Surveillance, Epidemiology, and End Results (SEER) program indicate that men aged 65 and older with distant metastatic prostate cancer experienced a year-over-year 0.4% improvement (95% CI 0.3-0.5) in three-year survival rates from 2004 to 2019 [2]. As men with prostate cancer live longer, there is a growing need for effective treatments with minimal toxicity, particularly for managing distant metastases.

Nonagenarians with advanced prostate cancer are often ineligible for cytotoxic therapies due to the heightened risks associated with these treatments. Prostate-specific membrane antigen (PSMA)-targeted radioligand therapies, which have demonstrated greater efficacy and a more favorable safety profile than docetaxel [3,4] and cabazitaxel [5], offer a promising alternative for patients with medical fragility due to advanced age or comorbidities [3,5]. Supporting this, a recent study of 80 octogenarians treated with Lu-177 PSMA-I&T reported a ≥50% reduction in prostate-specific antigen (PSA) levels in 46.3% of patients, with fewer than 10% experiencing significant toxicity [6]. Notably, other studies have suggested that taxane-naïve metastatic castration-resistant prostate cancer (mCRPC) patients undergoing Lu-177 PSMA therapy tend to have better survival outcomes and lower rates of adverse events compared to those previously treated with taxane-based chemotherapy [7]. This indicates that earlier integration of Lu-177 PSMA in carefully selected elderly patients may improve treatment tolerability and outcomes.

Here, we report on the promising outcomes of three nonagenarians with mCRPC who were deemed unsuitable for chemotherapy and treated with Pluvicto (Lu-177 PSMA-617/Lu-177 vipivotide tetraxetan), a PSMA-targeted radiopharmaceutical.

## Case presentation

This study complied with Health Insurance Portability and Accountability Act (HIPAA) regulations and was reviewed by an independent institutional review board (IRB). The analysis of retrospective, de-identified data was classified as exempt from IRB oversight under the Department of Health and Human Services regulations (45 CFR 46.104(d)(4)).

This retrospective case series included three nonagenarian patients with mCRPC who were treated with Lutetium-177 (Lu-177) vipivotide tetraxetan (Pluvicto, Novartis AG, Basel, Switzerland) between August 2022 and January 2024 at BAMF Health in Grand Rapids, Michigan, USA, following confirmation of PSMA-expressing disease on PSMA positron emission tomography (PET) imaging. While the retrospective analysis was conducted at a single institution (BAMF Health), the authors had additional affiliations, with two authors primarily affiliated elsewhere but also associated with BAMF Health at the time of data acquisition and manuscript preparation.

Eligibility criteria and contraindications

Life Expectancy Considerations

Patients were included only if a multidisciplinary assessment determined their estimated life expectancy was sufficient to complete at least one cycle of Pluvicto. Those with an anticipated survival of less than three months due to rapidly progressive disease or severe comorbidities were excluded.

Predisposing Toxicities

Patients with significant bone marrow suppression (e.g., grade 3-4 anemia, thrombocytopenia, or neutropenia), renal insufficiency (glomerular filtration rate <30 mL/min), or severe gastrointestinal symptoms that could affect treatment tolerance were excluded.

Prior Chemotherapy

While prior chemotherapy was not an absolute exclusion criterion, all patients in this series were deemed ineligible for taxane-based chemotherapy due to frailty, poor functional status, or pre-existing comorbidities such as cardiovascular disease, neurocognitive decline, or significant baseline cytopenias. This selection approach ensured that Pluvicto was administered to patients most likely to benefit from the treatment while minimizing undue burden.

For PSMA-PET/computed tomography (CT) imaging, patients received an injection of 111-259 MBq (3-7 mCi) of Gallium-68 (Ga-68) gozetotide (Illuccix®, Telix Pharmaceuticals Ltd., Melbourne, Australia) and were scanned 50-100 minutes post-injection using the uEXPLORER total-body PET/CT scanner (United Imaging Healthcare Co., Ltd., Houston, Texas, USA) with a PET acquisition time of three to five minutes. Imaging schedules varied: Patient 1 underwent scans before and after treatment, Patient 2 had a scan before treatment only, and Patient 3 had scans before and midway through treatment.

For PSMA-targeted radioligand therapy, patients received 200 mCi (±10%) of Lu-177 vipivotide tetraxetan per cycle, following the Pluvicto prescribing guidelines. Actual administered doses ranged from 188.3 to 205.6 mCi per cycle, with no dose reductions required. The number of treatment cycles was determined based on PSA changes, molecular tumor burden response via post-therapeutic single photon emission computed tomography (SPECT)/CT imaging (StarGuide™, GE HealthCare, Chicago, Illinois, USA), and evaluations of hematologic and chemistry parameters, as well as overall quality of life.

Whole-body SPECT/CT imaging was performed 20-24 hours post-infusion to assess Pluvicto biodistribution, tumor uptake, and volume. Both the 113 keV and 208 keV energy peaks of Lu-177 were acquired for analysis. PET/CT and SPECT/CT scans were manually annotated by trained non-physician specialists to segment PSMA-avid tumors. From these annotations, standardized uptake values (SUVmax and SUVmean) and total PSMA-avid tumor volumes were quantified across cycles to measure tumor burden and treatment response. The SUVindex was calculated by multiplying the SUVmean by tumor volume, while the tumor-to-background ratio was determined as SUVmax (tumor)/SUVmean (liver).

Biochemical progression was assessed according to the Prostate Cancer Working Group 3 (PCWG3) guidelines [8], with PSA progression defined as a ≥25% increase and an absolute rise of at least 2 ng/mL above the nadir, confirmed by a repeat measurement after a minimum of three weeks. Adverse events were graded using the Common Terminology Criteria for Adverse Events (CTCAE) v5.0 [9]. Baseline laboratory values were collected before the first treatment cycle, with additional evaluations conducted every two to three weeks during therapy.

Patient 1

Patient 1 was diagnosed with mCRPC four years prior and presented with biochemical progression, with a baseline PSA level of 119.3 ng/mL. His treatment history included one month of androgen deprivation therapy (leuprolide and bicalutamide), followed by 25 months of abiraterone. He later participated in the MK-641 clinical trial (NCT03834493), receiving pembrolizumab and enzalutamide/placebo. Additionally, he completed four cycles of radium-223 (Xofigo), with the last cycle administered 4.5 months before initiating Pluvicto at BAMF Health.

**Figure 1:**
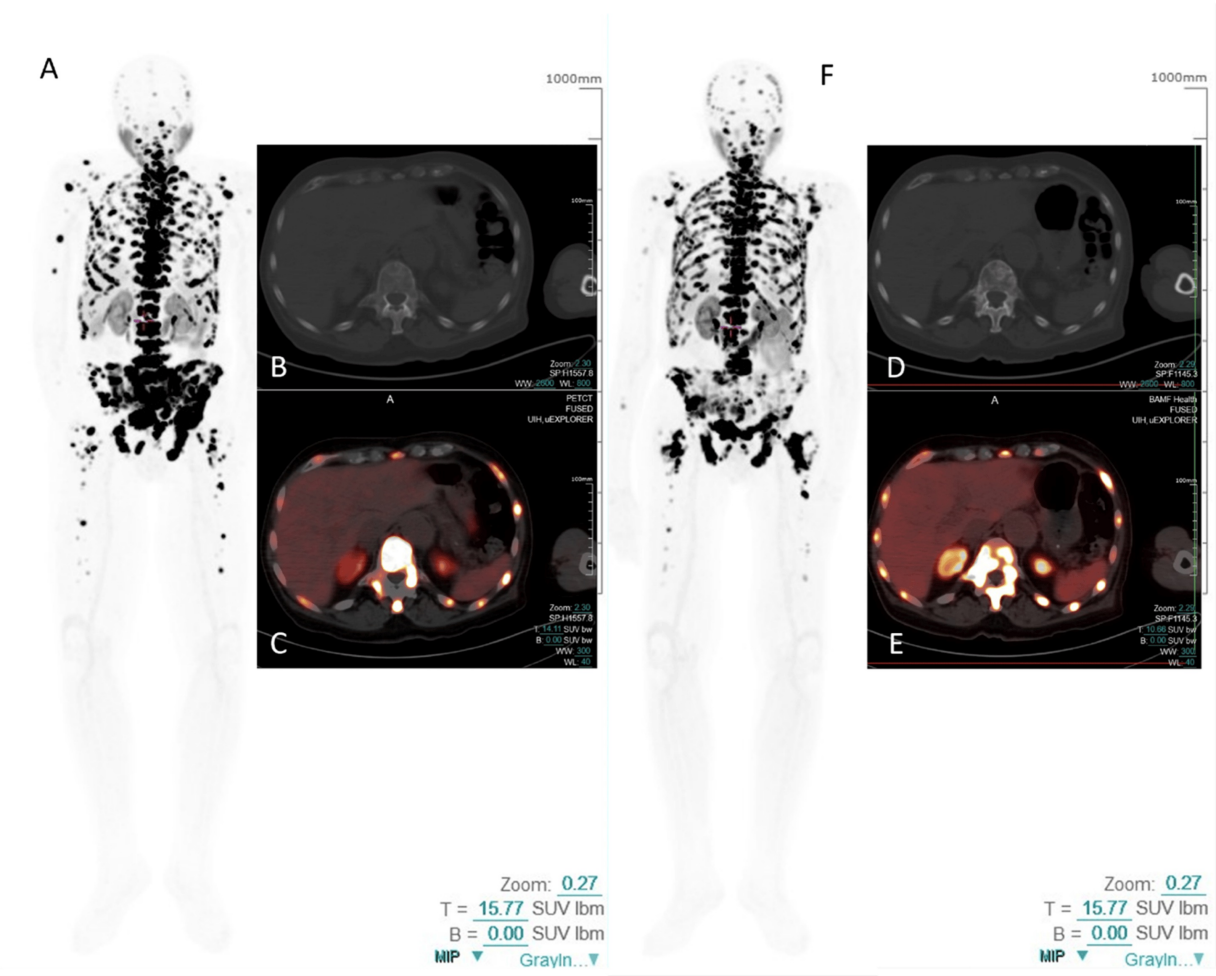
Pre- and post-therapy total-body PSMA-PET/CT images for Patient 1 The initial Ga-68 PSMA PET/CT (A, B, C) for Patient 1 revealed intense PSMA expression in extensive bone metastases throughout the axial and appendicular skeleton. Post-Pluvicto SPECT/CT imaging after the fourth cycle showed a mixed response among the metastases, along with significant biochemical disease progression, prompting a restaging Ga-68 PSMA PET/CT (D, E, F). This follow-up scan demonstrated an overall re-progression of the PSMA-positive molecular tumor burden. (A, F: MIP images; B, D: axial CT images; C, E: axial fused PET/CT images.) CT, computed tomography; PET, positron emission tomography; PSMA, prostate-specific membrane antigen; SPECT, single photon emission computed tomography

Despite a stable quality of life and managed pain under palliative care, the patient exhibited continued clinical and biochemical disease progression on radium-223, necessitating a transition to alternative systemic therapy. Initial PSMA-PET/CT imaging showed high PSMA expression in extensive osseous metastases throughout the axial and appendicular skeleton, with a tumor SUVmax of 114 (Figure 1). During Pluvicto treatment, he remained on androgen deprivation therapy with leuprolide, maintaining castrate testosterone levels.

**Figure 2:**
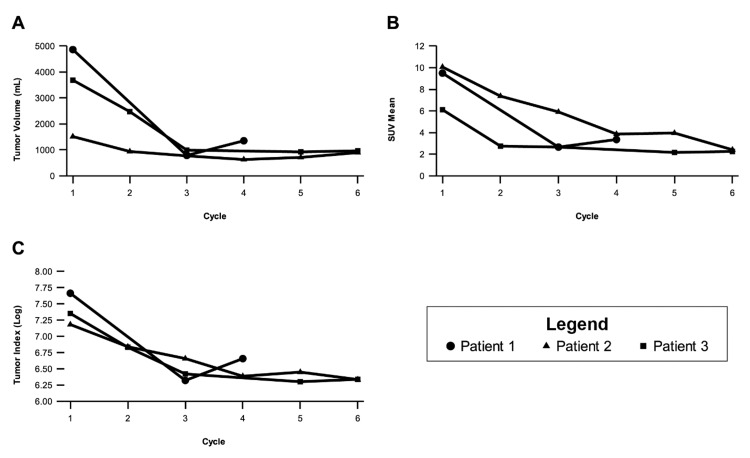
Radiographic tumor measurement changes per cycle as assessed by SPECT/CT (A) Change in SUVmean over the treatment course. (B) Change in tumor volume (mL) over the treatment course. (C) Change in tumor index (SUVmean * tumor volume in mL), with the Y-axis displayed on a log scale. All data points were obtained from 24-hour post-therapy SPECT/CT scans, except for the following: Patient 1 did not undergo SPECT/CT after cycle 2 due to a scheduling conflict, and his cycle 3 SPECT/CT was performed three days post-infusion. Patient 3 did not attend his 24-hour SPECT/CT after cycle 4. CT, computed tomography; SPECT, single photon emission computed tomography

Following the first Pluvicto infusion, SPECT/CT confirmed appropriate tracer uptake in all known metastases, with a tumor-to-background ratio of 93.41. Over the course of four Pluvicto cycles administered at six-week intervals, SPECT/CT demonstrated a 72% reduction in PSMA-positive tumor volume and a 65% decrease in the mean by cycle 4 (Figure 2).

**Figure 3:**
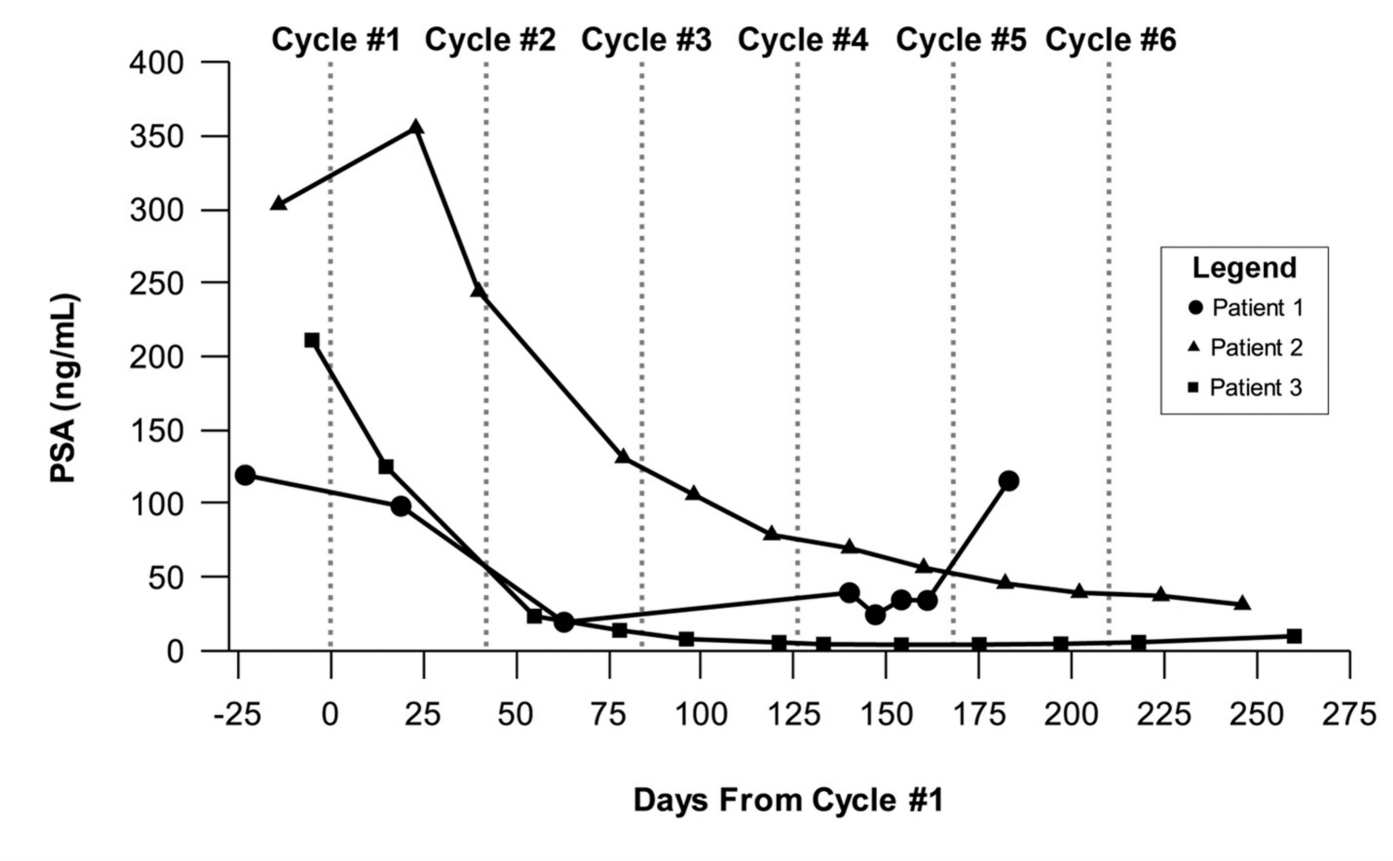
Changes in PSA blood levels throughout the treatment course This figure illustrates changes in PSA levels (ng/mL) over time relative to the number of days since the first Pluvicto injection. Vertical dashed lines indicate the approximate timing of each cycle, following the recommended six-week schedule, with a maximum of six cycles. Patients 2 and 3 completed all six cycles, while Patient 1 received four cycles. Day 0 corresponds to the first treatment cycle. PSA, prostate-specific antigen

PSA levels initially declined from 119.2 ng/mL to 98.2 ng/mL following the first cycle, reaching a nadir of 19.38 ng/mL after the second cycle. However, levels later increased, indicating biochemical progression according to PCWG3 criteria (Figure 3).

**Figure 4:**
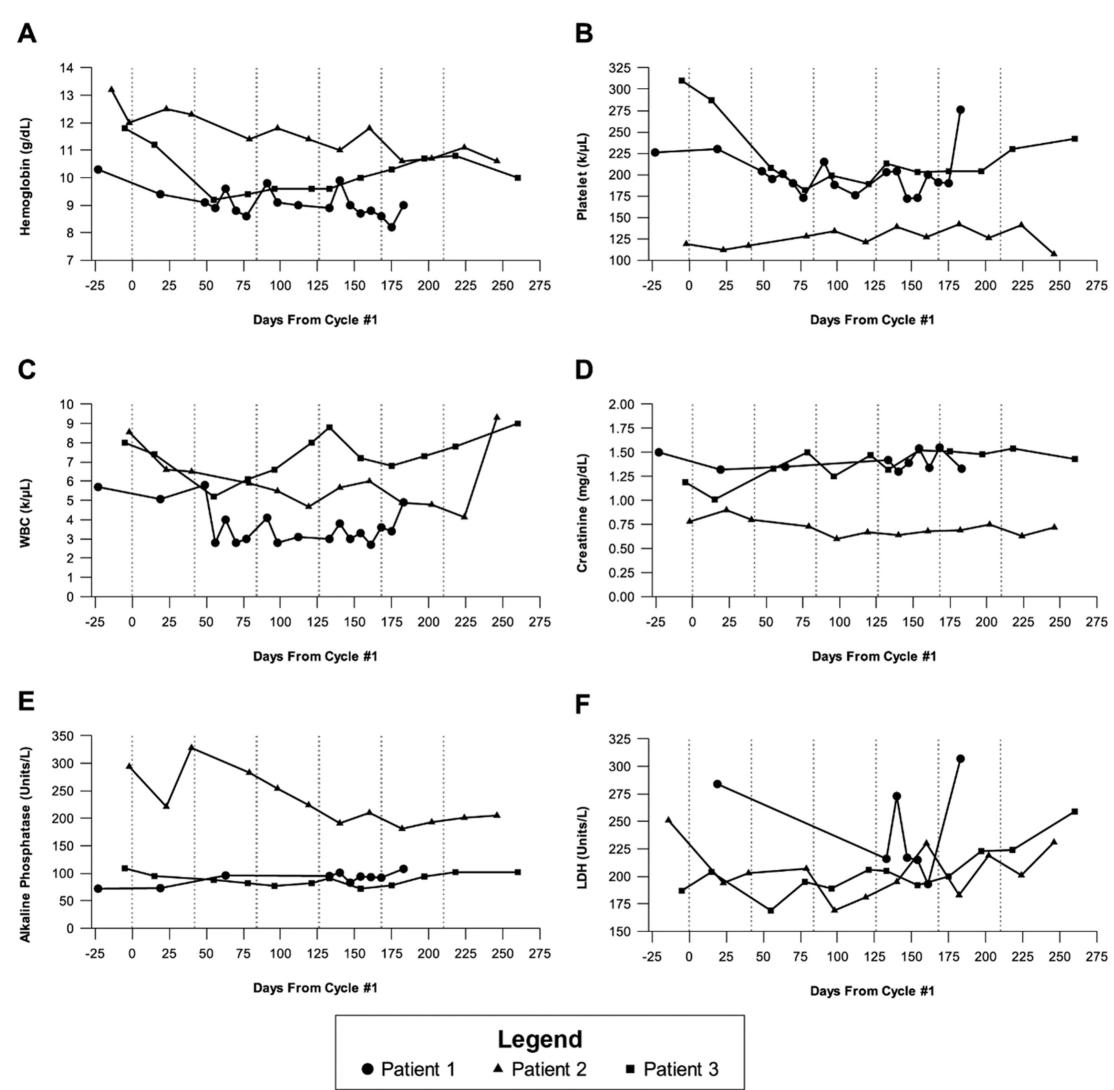
Changes in patient laboratory values over time (A) Hemoglobin. (B) Platelet count. (C) White blood cell count. (D) Creatinine. (E) Alkaline phosphatase. (F) Lactate dehydrogenase. Vertical dashed lines indicate the approximate timing of each cycle, following the recommended six-week schedule, with a maximum of six cycles. Patients 2 and 3 completed all six cycles, while Patient 1 received four cycles. Day 0 corresponds to the first treatment cycle.

Throughout treatment, the patient maintained an Eastern Cooperative Oncology Group (ECOG) performance score of 1 and experienced a 5% weight loss. Quality of life remained stable, with no reported symptoms of xerostomia, fatigue, or nausea. Mild hematologic changes were observed, including progression from grade 1 to grade 2 normocytic anemia and persistent grade 1 leukopenia following cycle 2 (Figure 4). Treatment was discontinued after four cycles due to disease progression, as restaging PSMA-PET/CT revealed an 11% increase in PSMA-expressing tumor burden. PSA progression-free survival was 140 days. The patient passed away approximately 8.5 months after the final Pluvicto cycle.

Patient 2

Patient 2, diagnosed with stage 4 prostatic adenocarcinoma, underwent radical prostatectomy 24 years before initiating Pluvicto. Biochemical recurrence occurred seven years prior to treatment, with rising PSA levels despite androgen deprivation therapy and subsequent enzalutamide, which was discontinued due to disease progression and poor tolerance. Chemotherapy was contraindicated due to advanced age and comorbidities, including a history of myocardial infarction and peripheral sensory neuropathy.

Baseline PSMA-PET/CT revealed intensely PSMA-positive osseous and mediastinal lymph node metastases, with an SUVmax of 51.70 in the lymph nodes. Leuprorelin was continued throughout Pluvicto therapy. Post-infusion SPECT/CT confirmed significant tracer accumulation, with a tumor-to-background ratio of 103.86. Over six treatment cycles, PSMA-positive tumor volume decreased by 41%, while SUVmean declined by 76% (Figure 2).

PSA levels initially rose from 303.00 ng/mL pre-treatment to 355 ng/mL after the first cycle, likely due to a PSA flare, but subsequently declined by 88%, reaching 37.3 ng/mL three weeks after the final cycle (Figure 3). At the six-week follow-up, no biochemical progression was observed, with PSA progression-free survival exceeding 252 days.

The patient completed all six cycles without a decline in quality of life or the development of grade 3 or 4 toxicities. Persistent grade 1 thrombocytopenia, mild anemia, and elevated alkaline phosphatase levels were observed throughout treatment (Figure 4). Xerostomia (grade 1) emerged in cycle 3 and persisted through post-treatment follow-up.

Patient 3

Patient 3 was diagnosed with stage 4 prostatic adenocarcinoma three years before initiating Pluvicto. At baseline, PSA was 489.6 ng/mL, with a biopsy confirming a Gleason score of 10. Imaging revealed metastases to the bone, lymph nodes, and lungs. Initial treatment included leuprorelin, enzalutamide, and denosumab, followed by abiraterone and prednisone after disease progression.

PSMA-PET/CT demonstrated extensive metastatic lesions with high radiotracer uptake. Given the patient’s advanced age and comorbidities, chemotherapy was deemed unsuitable, leading to the initiation of Pluvicto. SPECT/CT confirmed significant tracer accumulation, with a tumor-to-background ratio of 32.93. Over six treatment cycles, PSMA-positive tumor volume decreased by 74%, while SUVmean declined by 63% (Figure 2).

PSA levels dropped from 211 ng/mL pre-treatment to 4.04 ng/mL between cycles 4 and 5, with a nadir below 6 ng/mL (Figure 3). Post-treatment, PSA levels increased slightly but remained low. Biochemical PSA progression-free survival was estimated at 260 days.

The patient experienced transient grade 2 anemia and grade 1 creatinine elevation (Figure 4). Improvements in breathing and dysphagia were observed during treatment, along with an overall enhancement in quality of life. ECOG performance status improved from 2 to 1 before cycle 3. Persistent grade 1 xerostomia was the only notable long-term side effect following treatment.

## Discussion

The administration of Pluvicto to three nonagenarian patients with PSMA-positive metastatic prostate cancer resulted in notable responses, including significant reductions in PSA levels, SUV, and tumor volume. This is particularly significant given the lack of alternative systemic therapies available to these patients due to contraindications for chemotherapy and resistance to second-generation androgen receptor axis-targeting agents such as enzalutamide and abiraterone. Importantly, all three patients tolerated Pluvicto well without experiencing any grade 3 or 4 toxicities. However, treatment for Patient 1 was discontinued prematurely following the development of resistance by the fourth cycle. The efficacy, safety, and quality-of-life benefits observed in these patients underscore the need for further research into Pluvicto’s use in a demographic traditionally considered to have limited treatment options.

Evaluating the impact of cancer treatments on quality of life is crucial. In our study, one of the three patients demonstrated an improvement in ECOG performance status from baseline, highlighting Pluvicto’s potential to enhance functional status. The other two patients maintained their baseline ECOG scores throughout treatment - an encouraging outcome given their advanced age and extensive metastatic disease. Xerostomia, a common side effect, was observed in two patients at CTCAE v5.0 grade 1, with one case temporarily escalating to grade 2. Notably, none of the patients experienced nausea or vomiting that negatively affected their quality of life, suggesting favorable tolerability. Additionally, compared to treatments involving cabazitaxel, Pluvicto was associated with a 30% reduction in the incidence of grade 3 or 4 adverse events [5].

Administering cancer treatments to older patients is often challenging due to increased susceptibility to side effects [10]. Pluvicto, administered once every six weeks, offers greater convenience than chemotherapy regimens such as docetaxel and cabazitaxel, which require dosing every three weeks [5,11]. The reduced side effect burden and extended intervals between Pluvicto cycles minimize the need for complex travel arrangements, provide more time for mental and physical recovery, and allow patients to engage in activities that enhance their quality of life [5,6,12].

While Lu-177 PSMA therapy has demonstrated substantial efficacy in improving disease control and quality of life, emerging evidence suggests that patients who develop resistance to Lu-177 PSMA may still benefit from alternative PSMA-targeted radioligand therapies. Ac-225 PSMA, for instance, has shown promising results in patients with Lu-177 PSMA-refractory disease by leveraging the high linear energy transfer of alpha particles to enhance tumor control [13]. Similarly, Tb-161 PSMA, a novel beta-emitting radioligand, offers potential advantages by delivering both beta and Auger electrons, potentially increasing efficacy while minimizing toxicity [14].

This study has certain limitations, including a short follow-up period and a small sample size. To validate the promising findings observed, future research should involve larger cohorts and extended follow-up durations. Additional post-treatment analyses would enable comparisons with median survival rates from larger studies, such as the VISION trial, and other investigations, particularly those involving patients treated before taxane-based chemotherapy [15]. With encouraging results from the PSMA FORE study (NCT:04689828) supporting the use of Pluvicto before chemotherapy, more patients could benefit from this approach. Further studies focusing on nonagenarians will be necessary to evaluate Pluvicto’s role in pre-chemotherapy settings.

More frequent radiological assessments using SPECT/CT scans could enhance the understanding of disease progression beyond clinical evaluations and PSA levels. Conducting SPECT/CT scans after every cycle, rather than relying solely on pre- and post-treatment PET/CT scans, would allow for proactive disease management. This real-time data could facilitate individualized treatment modifications, moving away from the current one-size-fits-all approach to Pluvicto administration. However, the potential costs, radiation exposure, and logistical challenges associated with increased scanning frequency must be carefully weighed against the clinical benefits.

Moreover, combination radioligand therapy (RLT) strategies are emerging as a promising avenue for optimizing treatment responses. Dual radionuclide approaches, such as Ac-225/Lu-177 PSMA combination therapy, have been explored for their synergistic effects in overcoming heterogeneous tumor resistance, potentially leading to improved therapeutic outcomes [16]. Similarly, Tb-161/Lu-177 PSMA therapy is being investigated for its potential to enhance radiation dose distribution while maintaining manageable toxicity profiles [17]. These advancements in PSMA RLT highlight the evolving treatment landscape for mCRPC and offer renewed hope for patients with limited therapeutic options.

## Conclusions

Treatment with Pluvicto appears to be a safe, well-tolerated, feasible, and effective alternative for nonagenarian patients with mCRPC who are ineligible for chemotherapy.
